# Dorsal subthalamic deep brain stimulation improves pain in Parkinson's disease

**DOI:** 10.3389/fpain.2023.1240379

**Published:** 2023-08-16

**Authors:** Asra Askari, Jordan L. W. Lam, Brandon J. Zhu, Charles W. Lu, Kelvin L. Chou, Kara J. Wyant, Parag G. Patil

**Affiliations:** ^1^ Department of Neurosurgery, University of Michigan, Ann Arbor, MI, United States; ^2^ Department of Biomedical Engineering, University of Michigan, Ann Arbor, MI, United States; ^3^ Department of Neurology, University of Michigan, Ann Arbor, MI, United States

**Keywords:** Parkinson’s disease, movement disorder society unified Parkinson’s disease rating scale (MDS-UPDRS), pain, deep brain stimulation, subthalamic nucleus

## Abstract

**Introduction:**

Inconsistent effects of subthalamic deep brain stimulation (STN DBS) on pain, a common non-motor symptom of Parkinson's disease (PD), may be due to variations in active contact location relative to some pain-reducing locus of stimulation. This study models and compares the loci of maximal effect for pain reduction and motor improvement in STN DBS.

**Methods:**

We measured Movement Disorder Society Unified PD Rating Scale (MDS-UPDRS) Part I pain score (item-9), and MDS-UPDRS Part III motor score, preoperatively and 6–12 months after STN DBS. An ordinary least-squares regression model was used to examine active contact location as a predictor of follow-up pain score while controlling for baseline pain, age, dopaminergic medication, and motor improvement. An atlas-independent isotropic electric field model was applied to distinguish sites of maximally effective stimulation for pain and motor improvement.

**Results:**

In 74 PD patients, mean pain score significantly improved after STN DBS (*p* = 0.01). In a regression model, more dorsal active contact location was the only significant predictor of pain improvement (*R*^2^ = 0.17, *p = *0.03). The stimulation locus for maximal pain improvement was lateral, anterior, and dorsal to that for maximal motor improvement.

**Conclusion:**

STN stimulation, dorsal to the site of optimal motor improvement, improves pain. This region contains the zona incerta, which is known to modulate pain in humans, and may explain this observation.

## 1. Introduction

Pain is a common and distressing non-motor symptom of Parkinson's disease (PD), affecting up to 85% of patients and impacting quality of life (1). Subthalamic nucleus deep brain stimulation (STN DBS) improves global pain scores from 28% to 84% (2). However, the structure-function relationships underlying this finding remain unclear. Several studies correlate motor improvement and pain relief, while other studies find no relationship between motor and pain improvement after DBS (3–5).

Recent electrophysiologic studies have suggested distinct roles of STN DBS on pain and motor improvement (2, 6–8). Connections between STN subregions and brain areas involved in pain processing (2, 9–12). The proximity of the motor-optimal DBS stimulation locus to the zona incerta (ZI), a potential target for pain relief (13, 14), motivated us to investigate the role of active contact location on pain in PD patients undergoing STN DBS.

In this study, we evaluated the impact of active DBS contact location on changes in the MDS-UPDRS Part I pain score following STN DBS. We hypothesized, based on earlier work (14), that stimulation of the dorsal STN/zona incerta (ZI) could improve pain scores.

## 2. Methods

### 2.1. Participants

This retrospective observational study included patients with idiopathic PD who underwent STN DBS at the University of Michigan between 2009 and 2019 and completed the MDS-UPDRS Part I questionnaire. Written informed consent was obtained from all subjects. The study was approved by the Institutional Review Board (HUM00021058).

### 2.2. Surgical procedure

Several weeks before surgery, patients underwent a 3 T MRI to visualize the STN, using a validated, high-resolution protocol (15). On the day of surgery, patients were fitted with a Leksell stereotactic frame (Elekta Instruments AB, Stockholm, Sweden) and underwent a 1.5 T MRI. The 3 T MRI and 1.5 T MRI were co-registered using a mutual-information algorithm (Analyze 9.0; AnalyzeDirect, Inc, Overland Park, Kansas). The MR-visualized STN was then targeted, and localization was finalized with intraoperative microelectrode recording. During surgery, a movement disorders neurologist evaluated each patient intraoperatively for symptom improvement and side effect thresholds to optimize DBS lead placement. At the time of pulse generator implantation, 2–4 weeks after lead placement, a high-resolution CT scan (CT750 HD, GE Healthcare, Chicago, Illinois; 64-slice, 140 kV, 450 mA, 0.5 × 0.5 × 0.6 mm) was obtained to visualize electrode contacts after brain shift and pneumocephalus had resolved. DBS programming commenced 4–6 weeks after initial lead placement. Our detailed surgical protocol is described in a previous publication (16).

### 2.3. Active contact localization

Co-registered CT and MR images were oriented in Talairach space, relative to the midline and the intercommissural plane. The STN midpoint was defined as the point halfway between the STN oral and caudal poles (17), which were identified on coronal MRI. Coordinates of the active contacts were then determined and recorded relative to these MR-visualized STN midpoints. Lateral (X), anterior (Y), and dorsal (Z) directions relative to the STN midpoint were defined as positive.

### 2.4. Determination of loci of maximal effect

To determine the loci of maximal effect for motor improvement and pain relief, a weighted score was generated at each coordinate around the STN using the location of the active contact, the motor improvement score or the pain relief score, and the probability that the active contact would activate a neuron at the coordinate. An overall stimulation-weighted improvement score was assigned to each point in the STN region for each condition by summing across all patients. The simplex algorithm (MATLAB, MathWorks®, Natick, MA) was used to identify the locus associated with maximal motor or pain improvement. Spatial distributions (relative to STN midpoint in millimeters) and confidence intervals (CI) for maximal effect locations were calculated using the bootstrap technique. See Conrad et al. (16, 18) for greater detail.

### 2.5. Clinical assessments

Demographic information and levodopa-equivalent dose (LED) were collected. Clinical assessments included the MDS-UPDRS Part I pain score (item-9) and the MDS-UPDRS Part III (motor examination), which was measured before surgery in the OFF-medication condition (baseline) and 6–12 months later in the OFF-medication/ON-stimulation state. To complete the MDS-UPDRS Part I-9, patients were asked whether they have had any uncomfortable feelings in their body including pain, aches, tingling, or cramps over the past week, and then they scored from 0 (no uncomfortable feeling) to 4 (severe, that stoped them to do their daily activity of life) (19).

### 2.6. Statistical approach

An ordinary least squares regression model was used to examine the impact of active contact electrode location in each axis on pain score at follow-up while controlling for gender, age, LED change from baseline to follow-up, MDS-UPDRS-III percent change improvement, and pain score at baseline. Student's paired *t*-tests were used to determine if the post-DBS assessments significantly differed from baseline assessments. All analyses were 2-sided with a significance level of 0.05.

## 3. Results

### 3.1. Participants

Our sample population included 74 patients with idiopathic PD who underwent STN DBS (72 with bilateral implants, 2 with unilateral). Twenty-two (29.7%) patients were female. The mean age was 64.2 ± 7.7 years. There was a small but significant (*p *= 0.01) improvement from baseline pain (1.87 ± 1.16) to follow-up pain score (1.41 ± 1.07).

### 3.2. Relationship between pain relief and dorsal active contact location

Regression analyses were conducted separately for the left and right hemispheres with lead location predictors separated by axis, resulting in a total of 6 analyses [(right or left hemisphere) × (*X*, *Y*, or *Z*-axis)]. The results of the ordinary least square regression model revealed that age, LED change from baseline to follow-up, gender, and pain score at baseline were not significant predictors of pain score at follow-up in any model (*p* > 0.05).

**Figure 1:**
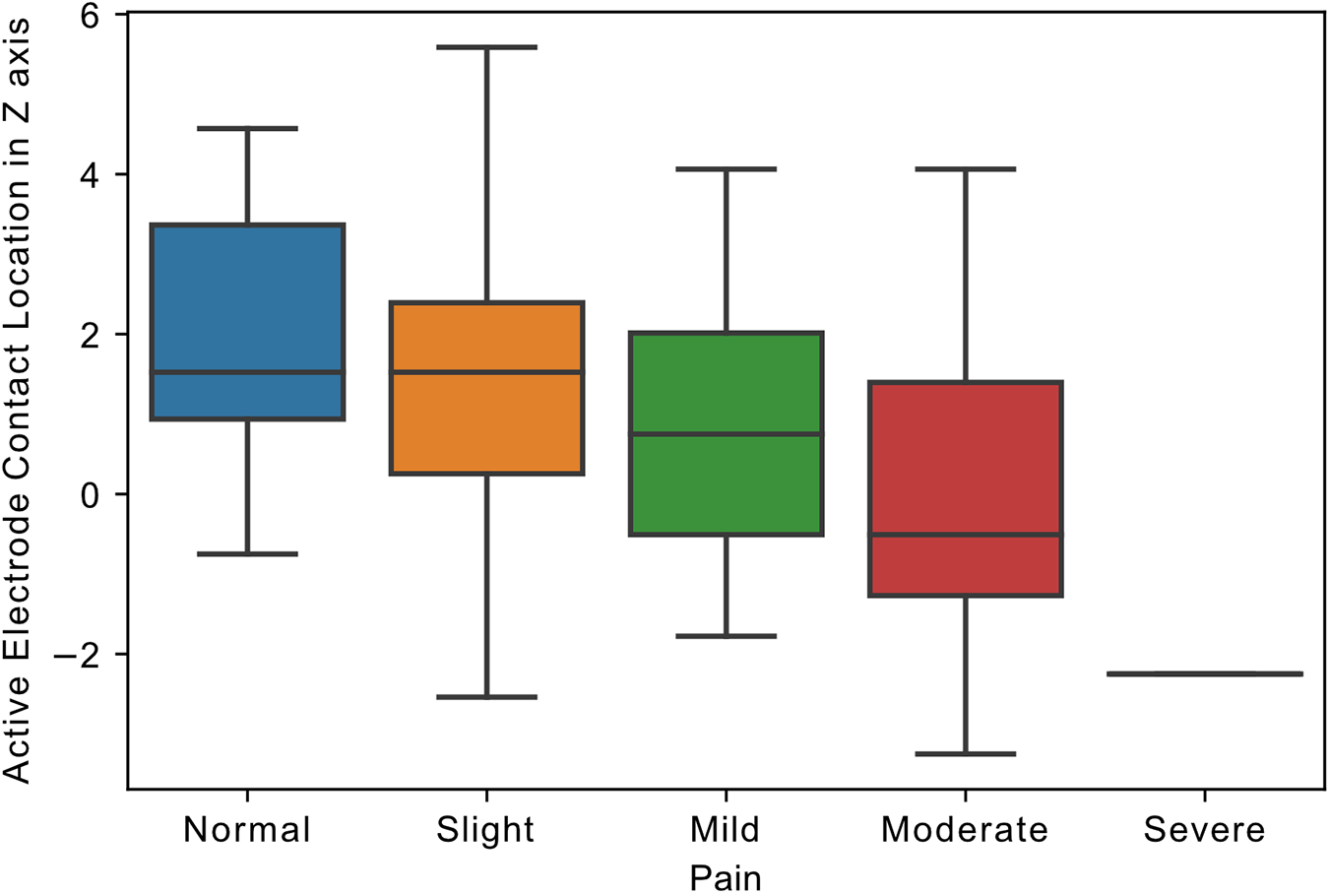
Relationship of active electrode location to pain. Boxplot, showing the distribution of follow-up pain scores and the distance of electrode active contact from STN midpoint in *z*-axis in the left hemisphere (in millimeters). Pain scores in MDS-UPDRS Part I ranged from 0 to 4, representing 0: Normal, 1: Slight, 2: Mild, 3: Moderate, and 4: Severe pain.

Active contact location in the *Z*-axis of the left hemisphere significantly predicted pain improvement (*β* = −0.2, *R*^2^ = 0.17, *p = *0.03), with dorsal location providing greater pain relief. By contrast, active contact location relative to the STN midpoint in the *X* and *Y*-axis in both hemispheres, and the *Z*-axis in the right hemisphere did not predict pain improvement (*p* > 0.05). In addition, the correlation coefficient between the pain follow-up score and left electrode active contact location in the *z*-axis was highly significant (*R* = −0.37, *p =* 0.001*;* Figure 1).

### 3.3. Loci of maximal motor and pain improvement

**Figure 2:**
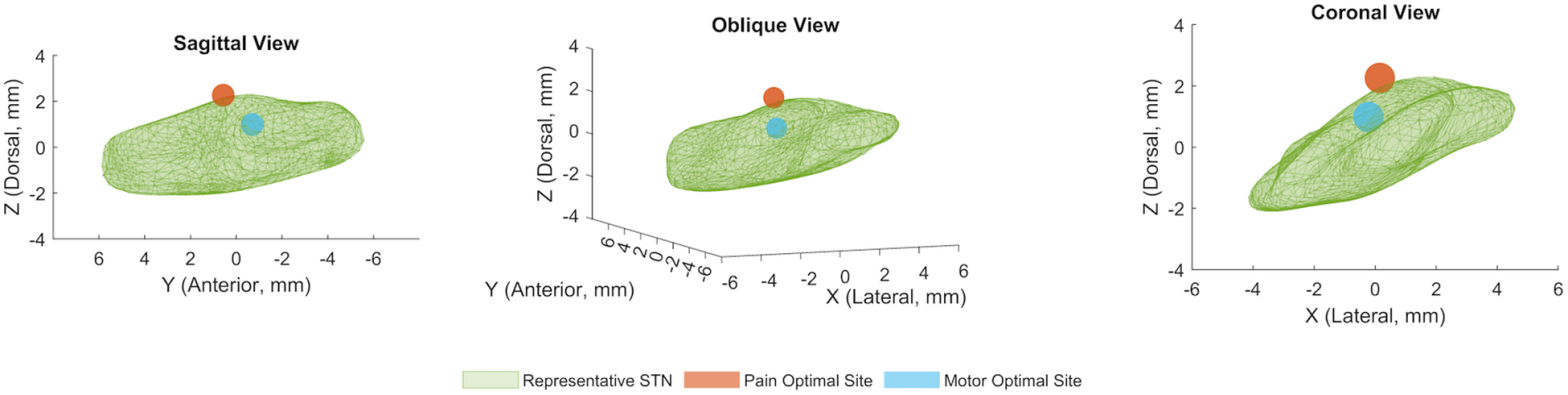
Locus of optimal pain and motor improvements in STN DBS. Mean coordinates for computational model-derived optimal sites of stimulation for pain and motor symptoms superimposed on a representative STN volume, in sagittal, oblique, and coronal views. The coordinates for the optimal site for pain are (*X* = 0.15 mm, *Y* = 0.57 mm, *Z* = 2.26 mm), and the coordinates for the optimal site for motor symptoms are (*X* = −0.22 mm, *Y* = −0.70 mm, *Z* = 0.98 mm) relative to the STN midpoint. All axis coordinates are in millimeters, and lateral, anterior, and dorsal are defined to be positive.

The locus of maximal effect for motor improvement was located medial [−0.22 mm, 95% CI (0.50, 0.04)], posterior [−0.70, (−1.10, −0.31)], and dorsal [0.98, (0.63, 1.34)] to the STN midpoint, while the locus of maximal pain relief was lateral [0.15, (−0.75, 1.18)], anterior [0.57, (−0.85, 2.03)], and dorsal [2.26, (1.23, 4.09)] to the STN midpoint (Figure 2). All coordinates are calculated in millimeters, with 95% confidence intervals, relative to the MR-visualized STN midpoint.

## 4. Discussion

We find that STN DBS significantly but modestly improves pain in PD patients, as measured by the MDS-UPDRS Part I. Remarkably, dorsal active contact location was the only significant predictor for improvement in pain, independent of age, gender, motor improvement, or LED changes. Moving from the ventral to the dorsal STN subregion was associated with decreasing pain; with the site of maximal pain relief lying lateral, anterior, and dorsal to the site of maximal motor improvement. There was no association between right-sided active contact location and pain score at follow-up. The optimal motor response was observed in left-sided and bilateral stimulation and not in right-sided stimulation (20). Likewise, the adverse effect of STN DBS was more significant for left-sided stimulation (21). Zhang and colleagues showed the laterality for nociceptive perception (22). However, additional study is needed to investigate pain perception laterality and the differential effects of left- and right-sided stimulation.

There are several detailed rating scales evaluating pain intensity and frequency in PD, thus recently the King's scale has been introduced as one of the most accurate (23). However, to our knowledge, there are no studies assessing the association between the STN subregions and a detailed pain rating scale in a large cohort. MDS-UPDRS part I-9 is not a detailed questionnaire. However, it has been validated (24) and is highly associated with quality of life (25).

Pain in PD is multifactorial, with evidence to support a nondopaminergic responsive central component (2, 26, 27). The role of ZI, the gray matter band located dorsal to STN, in central pain processing has been previously described (13, 28). Notably, the ventral subregion of ZI, enriched with GABAergic cells and connected to the spinothalamic tract and sensory thalamus, is an area involved in pain processing and modulation (29). We recently found that low-frequency, 20 Hz stimulation of zona incerta (ZI), modulates heat pain in humans (14). This finding supports the possibility of pain processing in ZI, specifically in PD patients. With the proximity of the dorsal STN subregion to ZI, we hypothesize that the spread of high-frequency current to the ZI ventral subregion could explain the positive effect of STN DBS on pain. The effect of low vs. high-frequency ZI stimulation on pain in PD needs to be addressed.

Neurostimulation of the dorsal STN subregion may also directly modulate pain processing. The functional connections between STN and pain processing regions in cortex, pedunculopontine nucleus, and parabrachial nucleus may play a role in this phenomenon (2). Additionally, STN local field potentials have been shown to respond to pain stimuli (30). However, the specific territories of STN involved in pain processing are not well understood. Future electrophysiologic studies could productively investigate the potential therapeutic role of the dorsal STN stimulation on pain.

While the results are intriguing, our study has several limitations. Most significant, our pain measure is limited to one item of the MDS-UPDRS-I. It is not possible from this measure to determine if DBS improves nociceptive or neuropathic pain. Future prospective studies are required to investigate the role of active contact location on improving different types of pain, through a prospectively applied, detailed pain battery such as the King scale (31). Another limitation is the simplified model of stimulation used. We applied an isotropic electric field model to localize the maximal effect site for pain improvement. However, modeling the non-isotropic volume of tissue activated (VTA) around active contacts could provide a more precise method to identify the optimal locus for pain improvement. Furthermore, without additional study, the specific overlap between the VTA and ventral ZI remains unknown. Future electrophysiologic studies and detailed imaging would be required to address this uncertainty. Finally, other components of Parkinson's disease, such as rigidity or dystonia, may contribute to patient pain, and the alleviation of these symptoms with STN DBS may result in pain relief, confounding our results. We have previously evaluated the locus of optimal relief of rigidity in STN DBS (18) and found it to be slightly, though not statistically significantly, medial, posterior, and *ventral* to the locus of overall optimal motor improvement. Hence, we believe that the locus of optimal pain improvement is distinct from that of motor improvements and also the secondary effects of motor improvement on pain relief. At the same time, further prospective study will be necessary to validate this hypothesis.

In conclusion, more dorsal stimulation in the STN region is associated with improvement of MDS-UPDRS part I-9, a validated rating measuring the impact of pain on quality of life. This may be related to stimulation in the vicinity of the ventral ZI, or direct pain-ameliorating effects on the dorsal STN. These findings motivate future studies to assess the effect of DBS active contact location on pain.

## Data Availability

The data analyzed in this study is subject to the following licenses/restrictions: These data are subject to Health Insurance Portability and Accountability Act (HIPPA) regulations. Anonymized data will be shared, subject to Institutional Review Board (IRB) approval. Requests to access these datasets should be directed to Parag G Patil, pgpatil@med.umich.edu

## References

[1] Valkovic P Minar M Singliarova H Harsany J Hanakova M Martinkova J Pain in Parkinson’s disease: a cross-sectional study of its prevalence, types, and relationship to depression and quality of life. PLoS One. (2015) 10(8):e0136541. 10.1371/journal.pone.0136541 26309254 PMC4550419

[2] Mostofi A Morgante F Edwards MJ Brown P Pereira EAC. Pain in Parkinson’s disease and the role of the subthalamic nucleus. Brain. (2021) 144(5):1342–50. 10.1093/brain/awab001 34037696 PMC7612468

[3] Defazio G Antonini A Tinazzi M Gigante AF Pietracupa S Pellicciari R Relationship between pain and motor and non-motor symptoms in Parkinson’s disease. Eur J Neurol. (2017) 24(7):974–80. 10.1111/ene.13323 28516474

[4] Silverdale MA Kobylecki C Kass-Iliyya L Martinez-Martin P Lawton M Cotterill S A detailed clinical study of pain in 1957 participants with early/moderate Parkinson’s disease. Parkinsonism Relat Disord. (2018) 56:27–32. 10.1016/j.parkreldis.2018.06.001 29903584 PMC6302227

[5] Nebe A Ebersbach G. Pain intensity on and off levodopa in patients with Parkinson’s disease. Mov Disord. (2009) 24(8):1233–7. 10.1002/mds.22546 19412949

[6] Zambito-Marsala S Erro R Bacchin R Fornasier A Fabris F Lo Cascio C Abnormal nociceptive processing occurs centrally and not peripherally in pain-free Parkinson disease patients: a study with laser-evoked potentials. Parkinsonism Relat Disord. (2017) 34:43–8. 10.1016/j.parkreldis.2016.10.019 27836714

[7] Marques A Chassin O Morand D Pereira B Debilly B Derost P Central pain modulation after subthalamic nucleus stimulation: a crossover randomized trial. Neurology. (2013) 81(7):633–40. 10.1212/WNL.0b013e3182a08d00 23864314

[8] Priebe JA Kunz M Morcinek C Rieckmann P Lautenbacher S. Electrophysiological assessment of nociception in patients with Parkinson’s disease: a multi-methods approach. J Neurol Sci. (2016) 368:59–69. 10.1016/j.jns.2016.06.058 27538603

[9] Gee LE Walling I Ramirez-Zamora A Shin DS Pilitsis JG. Subthalamic deep brain stimulation alters neuronal firing in canonical pain nuclei in a 6-hydroxydopamine lesioned rat model of Parkinson’s disease. Exp Neurol. (2016) 283(Pt A):298–307. 10.1016/j.expneurol.2016.06.031 27373204

[10] Hilker R Voges J Weisenbach S Kalbe E Burghaus L Ghaemi M Subthalamic nucleus stimulation restores glucose metabolism in associative and limbic cortices and in cerebellum: evidence from a FDG-PET study in advanced Parkinson’s disease. J Cereb Blood Flow Metab. (2004) 24(1):7–16. 10.1097/01.WCB.0000092831.44769.09 14688612

[11] Haynes WI Haber SN. The organization of prefrontal-subthalamic inputs in primates provides an anatomical substrate for both functional specificity and integration: implications for basal ganglia models and deep brain stimulation. J Neurosci. (2013) 33(11):4804–14. 10.1523/JNEUROSCI.4674-12.2013 23486951 PMC3755746

[12] Dellapina E Ory-Magne F Regragui W Thalamas C Lazorthes Y Rascol O Effect of subthalamic deep brain stimulation on pain in Parkinson’s disease. Pain. (2012) 153(11):2267–73. 10.1016/j.pain.2012.07.026 22964434

[13] Masri R Quiton RL Lucas JM Murray PD Thompson SM Keller A. Zona incerta: a role in central pain. J Neurophysiol. (2009) 102(1):181–91. 10.1152/jn.00152.2009 19403748 PMC2712264

[14] Lu CW Harper DE Askari A Willsey MS Vu PP Schrepf AD Stimulation of zona incerta selectively modulates pain in humans. Sci Rep. (2021) 11(1):8924. 10.1038/s41598-021-87873-w 33903611 PMC8076305

[15] Patil PG Conrad EC Aldridge JW Chenevert TL Chou KL. The anatomical and electrophysiological subthalamic nucleus visualized by 3-T magnetic resonance imaging. Neurosurgery. (2012) 71(6):1089–95; discussion 95. 10.1227/NEU.0b013e318270611f 22948201

[16] Conrad EC Mossner JM Chou KL Atlas-Independent PP. Electrophysiological mapping of the optimal locus of subthalamic deep brain stimulation for the motor symptoms of Parkinson disease. Stereotact Funct Neurosurg. (2018) 96(2):91–9. 10.1159/000486643 29791914

[17] Houshmand L Cummings KS Chou KL Patil PG. Evaluating indirect subthalamic nucleus targeting with validated 3-tesla magnetic resonance imaging. Stereotact Funct Neurosurg. (2014) 92(6):337–45. 10.1159/000366286 25358805

[18] Mossner JM Chou KL Maher AH Persad CC Patil PG. Localization of motor and verbal fluency effects in subthalamic DBS for Parkinson’s disease. Parkinsonism Relat Disord. (2020) 79:55–9. 10.1016/j.parkreldis.2020.08.023 32866879

[19] Goetz CG Tilley BC Shaftman SR Stebbins GT Fahn S Martinez-Martin P Movement disorder society-sponsored revision of the unified Parkinson’s disease rating scale (MDS-UPDRS): scale presentation and clinimetric testing results. Mov Disord. (2008) 23(15):2129–70. 10.1002/mds.22340 19025984

[20] Schulz GM Hosey LA Bradberry TJ Stager SV Lee LC Pawha R Selective left, right and bilateral stimulation of subthalamic nuclei in Parkinson’s disease: differential effects on motor, speech and language function. J Parkinsons Dis. (2012) 2(1):29–40. 10.3233/JPD-2012-11049 23939406

[21] Lueken U Schwarz M Hertel F Schweiger E Wittling W. Impaired performance on the Wisconsin card sorting test under left - when compared to right-sided deep brain stimulation of the subthalamic nucleus in patients with Parkinson’s disease. J Neurol. (2008) 255(12):1940–8. 10.1007/s00415-009-0040-1 19153637

[22] Zhang H Lu X Bi Y Hu L. A modality selective effect of functional laterality in pain detection sensitivity. Sci Rep. (2021) 11(1):6883. 10.1038/s41598-021-85111-x 33767243 PMC7994376

[23] Perez-Lloret S Ciampi de Andrade D Lyons KE Rodriguez-Blazquez C Chaudhuri KR Deuschl G Rating scales for pain in Parkinson’s disease: critique and recommendations. Mov Disord Clin Pract. (2016) 3(6):527–37. 10.1002/mdc3.12384 30363588 PMC6178703

[24] Gallagher DA Goetz CG Stebbins G Lees AJ Schrag A. Validation of the MDS-UPDRS part I for nonmotor symptoms in Parkinson’s disease. Mov Disord. (2012) 27(1):79–83. 10.1002/mds.23939 21915909

[25] Skorvanek M Rosenberger J Minar M Grofik M Han V Groothoff JW Relationship between the non-motor items of the MDS-UPDRS and quality of life in patients with Parkinson’s disease. J Neurol Sci. (2015) 353(1-2):87–91. 10.1016/j.jns.2015.04.013 25918077

[26] Millan MJ. Descending control of pain. Prog Neurobiol. (2002) 66(6):355–474. 10.1016/S0301-0082(02)00009-6 12034378

[27] Naser PV Kuner R. Molecular, cellular and circuit basis of cholinergic modulation of pain. Neuroscience. (2018) 387:135–48. 10.1016/j.neuroscience.2017.08.049 28890048 PMC6150928

[28] Hu TT Wang RR Du Y Guo F Wu YX Wang Y Activation of the intrinsic pain inhibitory circuit from the midcingulate Cg2 to zona incerta alleviates neuropathic pain. J Neurosci. (2019) 39(46):9130–44. 10.1523/JNEUROSCI.1683-19.2019 31604834 PMC6855685

[29] Mitrofanis J. Some certainty for the “zone of uncertainty”? exploring the function of the zona incerta. Neuroscience. (2005) 130(1):1–15. 10.1016/j.neuroscience.2004.08.017 15561420

[30] Parker T Huang Y Gong C Chen Y Wang S Green AL Pain-induced beta activity in the subthalamic nucleus of Parkinson’s disease. Stereotact Funct Neurosurg. (2020) 98(3):193–9. 10.1159/000507032 32348997

[31] Chaudhuri KR Rizos A Trenkwalder C Rascol O Pal S Martino D King’s Parkinson’s disease pain scale, the first scale for pain in PD: an international validation. Mov Disord. (2015) 30(12):1623–31. 10.1002/mds.26270 26096067

